# Impact of Inclination of Girders and Columns on the Effort and Stability of Flat Bar Frames

**DOI:** 10.3390/ma16186284

**Published:** 2023-09-19

**Authors:** Jacek Abramczyk, Katarzyna Chrzanowska, Wiesław Bielak

**Affiliations:** 1 Department of Architectural Design and Engineering Graphics, Rzeszow University of Technology, Al. Powstańców Warszawy 12, 35-959 Rzeszów, Poland; d565@stud.prz.edu.pl; 2 Department of Structural Mechanics, Rzeszow University of Technology, Al. Powstańców Warszawy 12, 35-959 Rzeszów, Poland; wbielak@stud.prz.edu.pl

**Keywords:** flat rod frames, structural bar systems, computer simulations, strength and stability, sheds, shape changes

## Abstract

The article describes a specific method of using innovative transverse systems of flat bar frames as structures forcing elastic shape transformations of nominally flat folded sheets into the forms of ruled shell roof coverings. An innovative method for parametric shaping these forms and arrangement of frames constituting structural systems of sheds with folded thin-walled roof coverings, taking account of the specificity of designing elastically transformed roof sheeting, was proposed. The proposed method for defining the loads of the considered frames supporting lower shelves of the folds of transformed roof sheeting, as loads distributed uniformly along the length of the upper chord of a roof frame girder, is also an innovative approach. The above unconventional premises result in the innovative topic of the research presented in terms of checking the impact of changing the shape of subsequent flat frames (intended for the construction of sheds roofed with the transformed sheeting) on the geometric and mechanical properties of the members of these frames. For the defined loads and the proposed parameterization of the frame forms, an innovative set of conditions was developed to optimize their performance, and then a theoretical analysis of the observed dependencies was carried out. This analysis was performed in an unconventional, novel way using section modules of the cross-sections of all members. The performed computer simulations confirmed the significance of changes in the inclination of girders and columns on the geometric and mechanical properties of the members. The obtained results are the basis and justification for simulations and tests in the scope of further modification of the form, loads, work, and methods of using various configurations of flat frames in constructions.

## 1. Introduction

Flat bar frames are very useful as structural systems supporting transversely thin-walled roof coverings [1,2]. Diversification of forms of the transverse frame systems arranged subsequently along the length of a building allows one to force different mutual positions of the roof girders or façade columns of these systems. A flat strip made up of many nominally flat thin-walled folded sheets can be transformed into various shell forms as a result of mutual inclination of roof girders or façade columns belonging to the subsequent flat frames [3].

**Figure 1:**
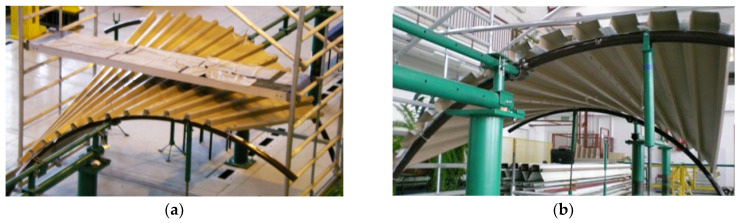
Two symmetric experimental corrugated shells supported by curvilinear skew directrices: (**a**) view from the top, (**b**) view from the bottom.

**Figure 2:**
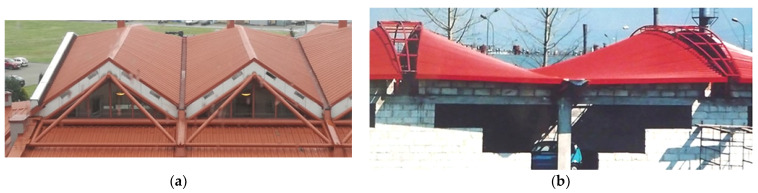
Roof sheeting composed of repetitive folded shell units and its structural system: (**a**) external view, (**b**) internal view.

The upper chords of roof girders of the frame systems are directrices supporting the lower flanges of all roof sheeting folds. Subsequent folds attached to the mutually skew directrices or poles change their shapes from folded flat into folded shell, so their bending, torsion or bending-torsion deformation is achieved [4] (Figure 1 and Figure 2). The arrangement of the shell folds, belonging to transformed sheeting, along the upper chord of a lattice girder and the support of the fold’s ends by the chord cause that all external loads are not individual forces concentrated at girder joints, but they are uniformly distributed load along the entire upper chord. A similar situation occurs in the case of column loads.

Inclination of girders or columns belonging to flat frames causes quantitative and qualitative changes in their structural work under the impact of the external loads. An intentional mutual inclination of adjacent directrices or columns belonging to subsequent frames changes the geometrical and mechanical properties of the thin-walled sheeting. By changing the shape of the subsequent transverse flat frames in a structural system, we can obtain an unconventional, interesting shell form of a roof or façade envelope [5,6]. However, the transformation causes an initial effort of the roof or envelope [7]. The article presents the impact of changes in the forms of the flat frames constituting structural systems of sheds on the effort, stability, displacement of joints, and deflection of the essential elements of these systems [8].

## 2. State of the Art

Metal frames are willingly used for building constructions due to their relatively low weight and freedom of shaping complex forms of roofs and facades. Many innovative methods for designing diversified unconventional building forms and their structural systems are presented in the monograph by Abdel et al. [9]. Effective, innovative building forms resulting from the rational work of their constructions are dealt with in the field of structural morphology [10,11]. The precursors of this field of knowledge are Motro [12] and Webster [13]. Smart materials used for members of the structural systems are very important to obtain their effective performance [14].

Geometrical and mechanical properties of nominally flat thin-walled corrugated sheets allow them to undergo large torsional and transverse bending deformations. If we assume that freedom of fold’s deformations is assured while fixing them on roof directrices, it is possible to adjust the bottom flanges of the shell folds to the various shapes and mutual positions of the directrices shaped [15] (Figure 2a,b).

The orthotropic mechanical properties of the nominally flat thin-walled folded metal sheets resulting from their folded geometric shape significantly restrict the freedom in shaping curved free forms of façades and roofs shallow parabolic-hyperbolic sheeting to limited by closed spatial quadrangles [16]. Reichhart exceeded the limitations related to the creation of only shallow and little-diversified hypars [4]. An appropriate technique for arranging the nominally flat folded sheets along the roof directrices requires calculating an optimum length of these directrices so that the freedom of the transverse shell fold’s deformations is developed. The freedom leads to effective mechanical work of each shell sheeting under a characteristic load. The spacing of the supporting points of all shell folds along each directrix must be adjusted to the diversified changes in the fold’s width of each transformed roof sheeting. Reichhart’s method makes it possible to shape many desired rational forms of roofs or facades by means of the appropriately developed shape, length, and mutual position of subsequent directrices arranged along the length of building structural systems.

A significant modification of Reichhart’s method was made by Abramczyk [17], who developed an algorithm for modeling the contraction appearing in each folded sheet subjected to torsional or bending-torsional transformation with the help of the lines of striction of various ruled surfaces. Abramczyk and Chrzanowska elaborated a new approach to design several properly correlated directrices to enforce the required shapes of the entire transformed folded sheeting [18]. The achieved forms and performance of their structural systems show the decisive influence of the mutual distance and inclination of all skew directrices belonging to the designed structural systems on the form, effort, and stability of these systems. Thus, a holistic method for shaping plane transverse structural systems containing mutually skew directrices forcing various attractive novel forms of the designed folded roof and elevation sheeting is needed.

In general, the static-strength performance of the common spatial and planar structural systems is described by Zhang et al. [19], and Martin and Purkiss [20]. Main problems related to shaping flat frames as structural systems are presented by Lam et al. [21]. The subsequent frames positioned along the length of a designed building can be of various forms [22]. Geometric and strength properties, as well as the stability of the systems supporting the transformed folded shell roofs, are outlined by Gomes et al. [23]. Ram and Gupta [24] and Somma and Vit [25] present the main issues related to shaping various metal trusses. Wadenier et al. [26] describe quantitatively and qualitatively static-strength work and the ability to maintain the stability of truss joints. Methods for designing stable plane structural systems are presented by Qu et al. [27] and Ziemian et al. [28].

The structural performance of tubular bar structures is also presented by Marshal [29]. Significant factors employed for shaping rational structural systems, i.e., strength work and stability of individual elements, their joints, and entire structure, were defined by Ziemian et al. [28] and Jarmai and Farkas [30]. The local stability of members is considered in detail by Hillebrand et al. [31].

All permissible loads and their combinations must be taken into account in each design process of buildings and their structural systems. Various directions of loads, including vertical, horizontal, and normal to the wall or roof surfaces, affect the complex equilibrium conditions of joints and members Kurobane et al. [32] and Packer and Henderson [33].

## 3. Aim

The aim of the research is to analyze the impact of changes in the inclination of a number of the selected elements of flat bar structural systems supporting various transformed thin-walled folded sheeting on the overall stability and the change in the strength properties of the elements belonging to these systems. A basic configuration of the simulated flat frame systems consists of a horizontal lattice girder and two vertical columns. Several planar lattice systems derived from the basic configuration have been created as the result of the inclination of their columns to the vertical or their girders to the horizontal. Computer models of the above derivative and basic rod systems were shaped to simulate their structural performance using the incremental non-linear Finite Element Method. The performed simulations allow one to observe a few major trends in changes in their strength work and ability to maintain overall stability.

## 4. Methodology

**Figure 3:**
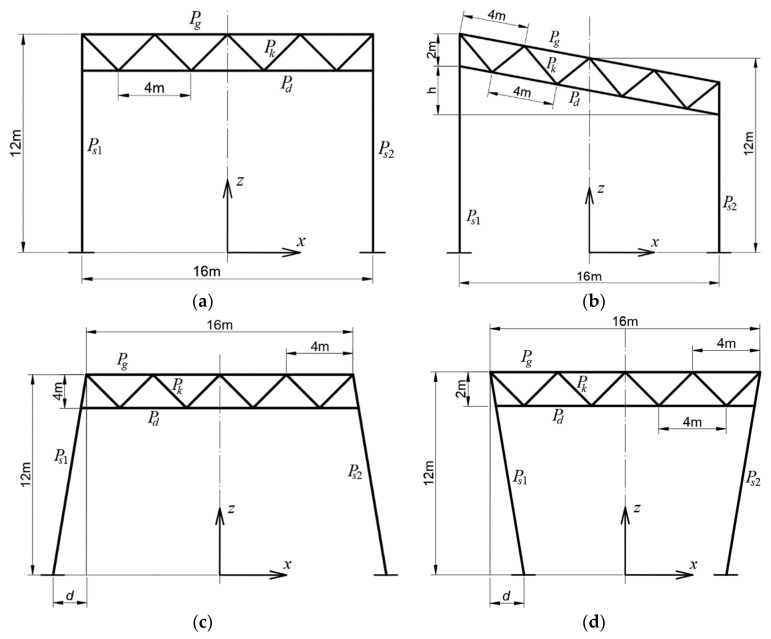
The schemes of four different types of the tested flat frames consisting of single-branch columns Ps1 and Ps2, girders with parallel chords Pg and Pd, and cross braces Pk of type V: (**a**) the rectangular basic frame Kb0, (**b**) the rectangular-trapezial derivative frame Kg, (**c**) trapezial frame Kce, (**d**) the inverted trapezial derivative frame Kci.

Each analyzed flat bar transverse system consists of a lattice girder with a height of 2 m and two single-branch columns. The upper and lower chords of the girder are connected to each other with V-diagonals spaced every 4 m. Its girder is horizontal. The two vertical columns are 12 m high, and their spacing is equal to 16 m. The following four frame shape types were considered: rectangular (Figure 3a), rectangular-trapezial (Figure 3b), trapezial (Figure 3c), and inverted trapezial (Figure 3d), with the basic shape being rectangular. For each of the above four shapes, four arbitrary load schemes were assumed, described at the end of this section. Then, the size of the cross-sections of individual types of elements of these frames was calculated. The method of optimizing these cross-sections of each frame is also described later in this section.

In the beginning, arbitrary cross-sections of all elements of an initial basic configuration Kb0 were adopted. A basic frame configuration Kb was obtained as the result of an optimizing process of Kb0 loaded with the above-mentioned four types of uniformly distributed load. Based on Kb, several derivative configurations were created as follows.

The first type of derivative configurations are the Kg configurations characterized by girders tilted to a horizontal plane and vertical columns (Figure 3b). The second type of derived configurations is composed of the Kci and Kce configurations characterized by columns inclined to the vertical. The former one has columns tilted with their bases outward (Figure 3c) and the latter one tilted inward (Figure 3d).

The columns are fixed in the foundation. All rods of the same element of a respective frame configuration have identical cross-sections, except for the columns of each derivative combination Kg, where their poles vary in length.

The research is related to the execution of several computer simulations modeling the mechanical performance of the basic and derivative frame configurations, accomplishing optimization of the cross-sections of their individual elements. The models were made in the Robot program [34]. An elastic second-order analysis was used in the calculations to capture the increments of bar bending stiffness caused by their longitudinal forces and transversal stiffness of the whole frame. Finite Element Method was used to perform incremental nonlinear calculations to model and compute the geometric and mechanical characteristics of the optimal cross-sections of the examined transverse frame elements. Initial geometrical imperfections were not taken into account. It was assumed that the idealized model of each bar is a straight segment with an assigned cross-section taken from the library of the computer program profiles based on the code requirements [20,35]. The accuracy of the geometric modeling is 1 mm, and the accuracy of the strength calculations is 1 MPa.

The incremental non-linear calculation method allows the discrete load values to be increased in subsequent calculation steps. The decrease in the stiffness of the entire frame and its individual bars caused by longitudinal eccentric compression forces acting in the bars and forces oriented laterally in relation to the vertical position of a flat frame was taken into account. The displacements of joints and bending of frame elements (appearing due to the eccentricity of the longitudinal rod forces) were also taken into account.

The research carried out was divided into four main steps. In the first step, a basic framework configuration, Kb0, was optimized to achieve Kb. The arbitrary optimization condition is composed of three restrictions: (1) the maximum allowable effort of all individual bars equal to 235 MPa +/− 3% for S235 steel, (2) the cross-section class not higher than 3, (3) the maximum low value of the critical load factor determined for each frame configuration, but not lower than 1.

The results of the simulations accomplishing the initial concept adopted by the authors prior to the study and related to the optimization of only the Kbo basic rectangular frame configuration into Kb and then using the calculated cross-sections for the analogous elements of the subsequently simulated derivative configurations turned out to be impossible because of the very high stresses appearing in their columns and chords.

Therefore, the optimization processes were also carried out for all derivative configurations. In addition, instead of analyzing the trends in changes in the stress levels appearing in all bars, the trends in changes in the section modules of the optimized cross-sections and the optimal critical load factor, resulting from the change in lattice girder or pole inclination to a horizontal plane or the vertical, were analyzed. The section modules were considered without taking the area and moment of inertia of the cross-sections into account due to the substantial share of bending moments appearing in the tilted columns of the derived configurations.

In the second step of the research, computer models of flat frames characterized by different inclinations of their girders to the horizontal were built. Three different discrete values of the h parameter belonging to the set {1.5, 3.0, 4.5 m} were adopted (Figure 3b). Three configurations created in this manner were called Kgi (*i* = 1 to 3). They were loaded and optimized in the same way as the basic Kb configuration.

In the third step, several simulations of non-rectangular frame systems Kci and Kce derived from Kb were carried out. The specific property of these configurations consists in the inclination of their columns to the vertical at the same angle. Four different values of the d parameter were assumed for Kce (Figure 3c) and four for Kci (Figure 3d). The adopted values of the d parameter belong to the set {1, 2, 3, 4 m}. These configurations were loaded and optimized in the same way as Kb.

In the fourth last step of the research, several diagrams showing the effect of the inclination changes of each relevant frame element influencing the overall stability of the frame and the strength properties of its individual bars were developed.

As stated at the beginning of this section, for each of the simulated basic and derivative frame configurations, four different types of loads were assumed. These loads were taken as characteristic of sheds covered with thin-walled, transformed folded sheeting. The nature of the load acting on each frame results from the way each fold was fixed to the roof directrices. The upper chords of the examined frame girders constitute the roof directrices.

**Figure 4:**
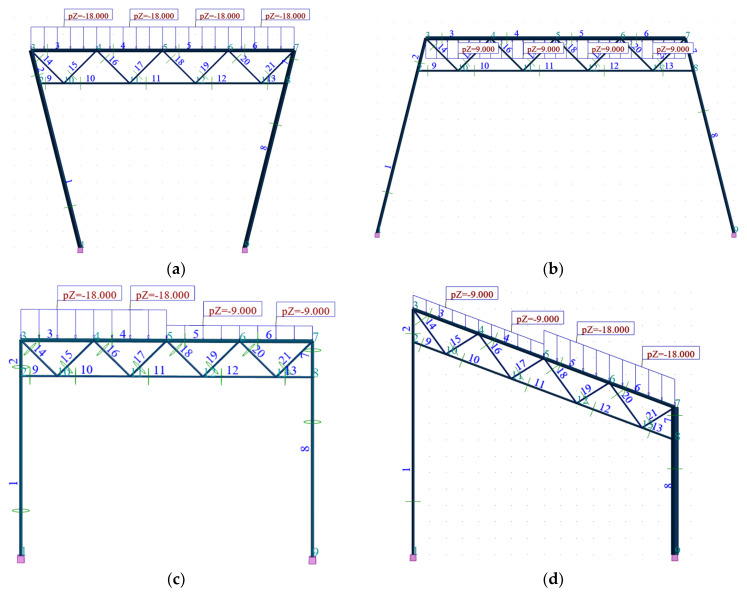
Four types of loads applied to each of the examined flat frame configurations: (**a**) the downward vertical load, (**b**) the upward load perpendicular to girder’s chord, (**c**) the unsymmetrical vertical load, (**d**) the unsymmetrical vertical load of applied to an unsymmetrical configuration.

The first type of load is a vertical load q = 18 kN/m^2^ uniformly distributed over the length of the top chord of each frame (Figure 4a). This value results from assuming a roof load of 3 kN/m² and a spacing of 6 m taken for the flat rod frames arranged along the shed’s length. The second and third types are loads ununiformly distributed along the length of the upper chord according to Figure 4b,c with the values q and q/2. The fourth type is the load perpendicular to the upper chord’s axis and pointing upwards, i.e., from the foundation side to the girder, evenly distributed along the length of the upper chord of the lattice girder (Figure 4b). Since the load corresponds to the wind suction acting on the shed roof, its value was assumed to be 1.5 kN/m^2^ ∗ 6 m, which is 9.0 kN/m. In addition, the self-weight of each frame was taken into account in each case of the simulations. Each of the presented load types was employed during the optimizing process of each derivative and basic frame configuration.

## 5. Results

The basic geometric and mechanical properties of the frame elements calculated as a result of the Kb’s optimization process are presented in Table 1. From the performed calculations, it follows that the examined upper chord P_g_, lower chord P_d_, and diagonals P_k_ are the most strengthened elements of the basic configuration Kb subjected to the adopted loads. The total resistance of the columns P_s_ cannot be used because the ability to maintain the general stability of the entire rectangular frame has a decisive impact on the size of their cross-sections. The optimized cross-sections of Kb were calculated as a result of many successive calculations consisting of changes in the cross-section sizes.

Opposite stress signs appearing in the same elements, and even in their bars, result from different values, directions, and senses of the four different types of loads mentioned above. The negative sign means tensile stresses and the positive sign means compressive stresses, according to the convention adopted in the computer program employed. The local stability of each bar is maintained by adopting a respective cross-section class for each element. The ability to maintain sufficient overall stability of each frame is defined by means of a critical factor that has to be greater or equal to 1.0.

The characteristics of the cross-sections calculated for Kb were then used to create the initial derivative configurations Kg0, Kce0, and Kci0. These initial derivative configurations were the basis for the calculations related to the optimization process of the derivative configurations Kgi (i = 1–3), Kcer (r = 1–4), and Kcij (j = 1–4). The results obtained for Kgi are given in Table 2.

The results obtained for the inverted trapezial derivative configurations Kcij (j = 1–4) are presented in Table 3. The results obtained for the trapezial derivative configurations Kcer (r = 1–4) are shown in Table 4.

## 6. Analysis

**Figure 5:**
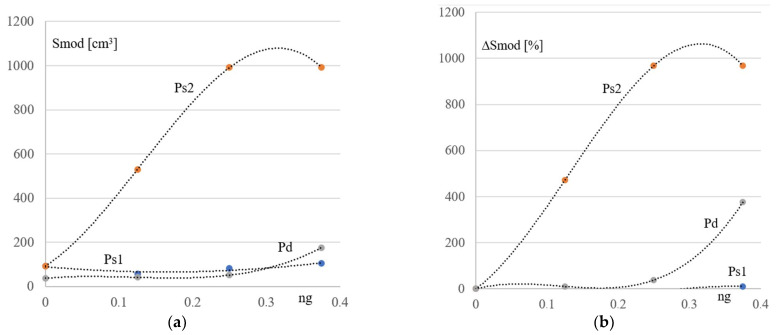
Three lines Ps1, Ps2, and Pd presenting three relations occurring between the values of the elastic section modules Smod calculated for the optimized cross-sections of the examined columns P_s1_, P_s2_, and bottom chord P_d_, and the inclination ng of the simulated lattice girders: (**a**) the absolute values of Smod, (**b**) the ΔSmod relative increments of Smod.

The analysis of the strength work and stability of the examined frames results in a comparison of section modules calculated for the optimal cross-sections of the most strengthened elements, such as the columns and bottom chords of the simulated basic and derivative configurations Kb and Kgi. Two diagrams shown in Figure 5a,b were built on the basis of these section modules, where three continuous lines present three relationships between the values of the above-mentioned section modules of the P_s1_ and P_s2_ columns and the bottom chord P_d_, and the inclination ng of the Kgi’s lattice girders. The ng slope is the ratio of the frame height h by the distance of 16 m between the columns of the rectangular basic frame configuration.

The dotted lines: Tend P_s1_, Tend P_s2_, and Tend P_d_ from Figure 5a illustrate three general trends in changes in the size of the optimal cross-sections of the above elements caused by the changes in the inclination of the considered frame girders to a horizontal plane. Absolute values of the section modules Smod calculated for the obtained optimal bar cross-sections of the simulated frame configurations are measured along the ordinate axis. The relative increments ΔSmod of these modules Smod are measured along the ordinate axis presented in Figure 5b. The girder inclination is measured along the axes of the abscissa.

**Figure 6:**
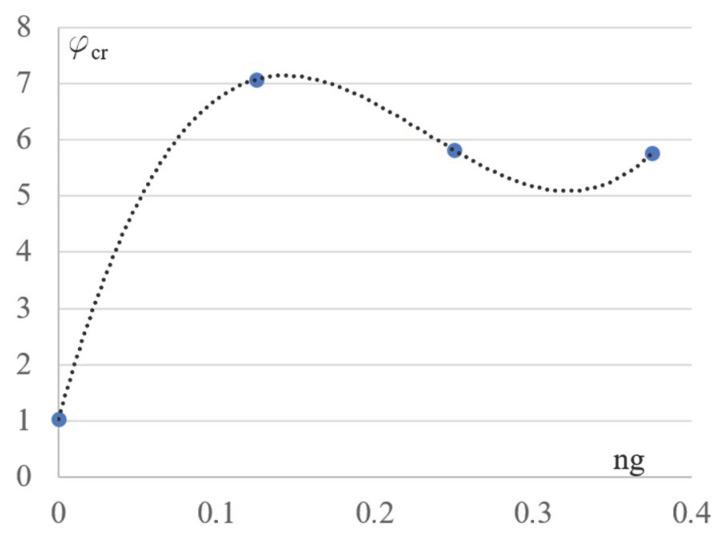
The dependence between the critical load factor φ_cr_ and the ng inclination of the girders.

**Figure 7:**
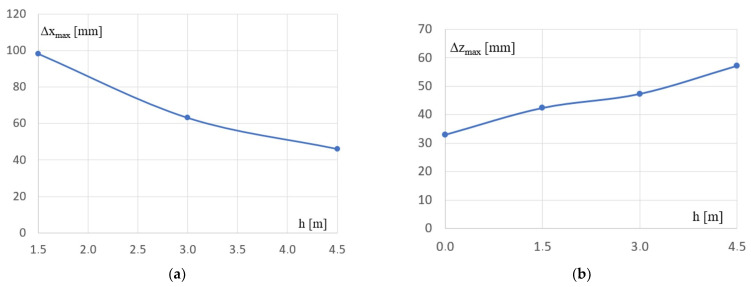
The relations occurring between the h difference in the height of the P_s1_ and P_s2_ columns of Kgi and the displacement of two selected frame joints: (**a**) the extremal horizontal displacement of the node 2 belonging to P_s1_, (**b**) the extremal deflection of the lattice girder (the displacement of the top chord’s node 5, see Figure 4).

The diagrams shown in Figure 5, Figure 6 and Figure 7 presented below were developed on the basis of the data given in Table 3 and Table 4. They illustrate the influence of the column’s inclination on the mechanical properties of the derivative frames Kgi with the tilted lattice girders.

In the above diagrams, the zero value of ng was assigned to the corresponding value of the elastic section modulus of the optimized cross-section calculated for each element of the basic rectangular frame configuration. The change in the course of any line from the above-mentioned diagrams illustrates the change in the section modulus of the corresponding element of the successively simulated derivative configurations. Significant changes in the course of the above-mentioned lines indicate a significant impact of the girder inclination on the mechanical properties of the examined frame.

In order to accurately illustrate the above-mentioned changes (and the above-mentioned impact), a diagram showing the impact of the girder inclination ng on the relative (percentage) increments ΔSmod in the value of the Smod section modulus of the optimized frames in relation to basic configuration frame was built (Figure 5b). These changes are very large and reach 1000% in relation to the elements of the basic rectangular frame with vertical poles (Figure 5b), which proves a very large influence of the girder inclination on the mechanical properties of the considered flat frames. Such a large impact results from the change in the nature of the framework. For example, the columns of the rectangular configuration are subjected to an almost axial load. However, they are bent in the examined derivative configurations.

Figure 5a,b shows: 1. a strong significant influence of the inclination of the examined lattice girders on the strength work of their lower columns—the Ps2 line, 2. the less significant influence of the inclination of the lattice girders on the strength work of the bottom chord—the Pd line, 3. the insignificant influence of the lattice girder inclination on the strength work of the higher column—the Ps1 line.

An important tendency to qualitative changes in the mechanical properties of the analyzed frame configurations is shown in Figure 6. Changes in the values of the critical load factor φ_cr_, caused by the change in the lattice girder slope, start with the value 1.04 and increase very fast. This value obtained during the optimizing process of the cross-sections of the Kb’s elements limits the effort of the compressed columns of the rectangular configuration to *σ*_c_ = 207 MPa. This value makes it impossible to exploit the total bearing capacity of the columns. Therefore, the overall stability of the frame plays an important role in the optimization of the Kb basic configuration. In the case of the examined derivative configurations Kgi, the decisive role in shaping the optimal cross-sections of all elements is played by their effort.

The frame girder slope causes an increase in φ_cr_ (Figure 6). Next, its values stabilize around the value 6 and do not change significantly with the increase of ng. Thus, we observed a significantly greater ability to maintain the overall stability of the derivative configurations Kgi than for the rectangular configuration Kb due to the special nature of the loads transmitted from the horizontal or inclined top chords to the vertical columns of different lengths.

Joint 2, see Figure 4a–d, is characterized by the extreme displacement Δx_max_ in the direction of the x axis (Figure 7a) among all joints of the Kb and Kgi configurations. On the other hand, the extreme deflection Δz_max_ occurs in the lattice girders of Kb and Kgi configurations. This is the deflection in the z-axis direction.

**Figure 8:**
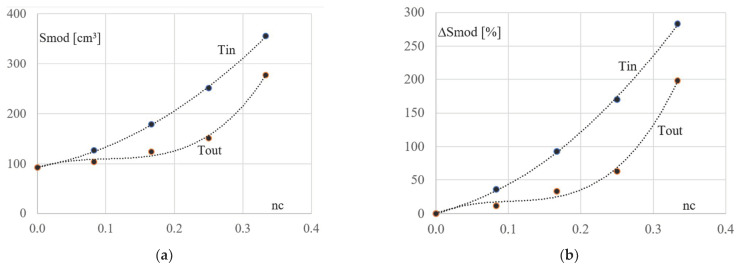
The Tin and Tout lines showing the relationships between the Smod section modules of the column cross-sections, the inverted trapezial Kcer (r = 1–4) configurations, and the trapezial Kcij (j = 1–4) configurations, respectively, and the inclination nc of these columns: (**a**) the absolute values of the Smod section modules, (**b**) the ΔSmod relative increments of Smod.

**Figure 9:**
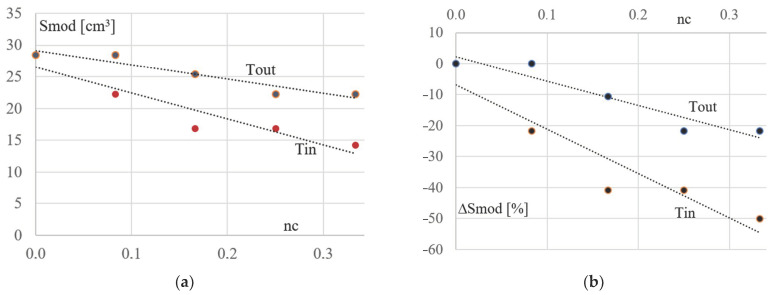
The Tin and Tout lines showing the relationships between the Smod section modules of the girder diagonal cross-sections of the inverted trapezial Kcer (r = 1–4) and trapezial Kcij (j = 1–4) configurations, respectively, and the inclination nc of their columns: (**a**) the absolute values of the Smod, (**b**) relative increments ΔSmod.

**Figure 10:**
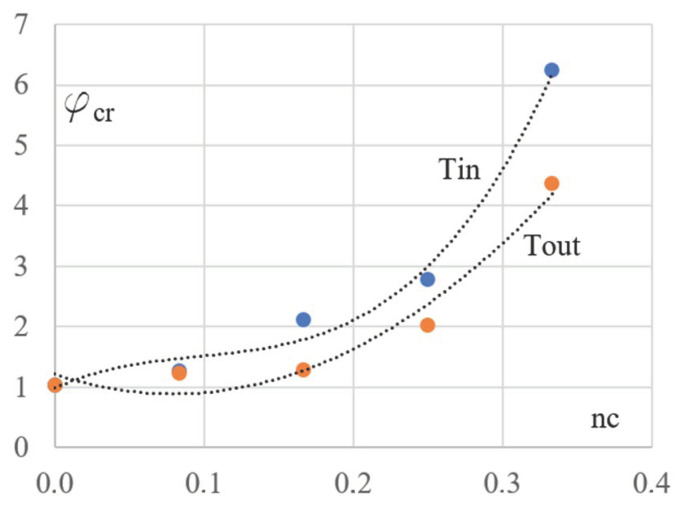
Two dependences of the critical load factor φ_cr_ on the nc column slope calculated for the inverted trapezial configurations Kcer (r = 1–4)—the Tout line and the trapezial configurations Kcij (j = 1–4)—the Tin line.

The following diagrams presented in Figure 8, Figure 9 and Figure 10, created on the basis of the other data given in Table 3 and Table 4, also show an influence of the column’s inclination on the mechanical properties of the derivative trapezial frames Kcij and Kcer.

The second type of the considered derivative configurations are frames characterized by the inclination of their columns to the vertical. For the optimized cross-sections of columns and diagonals of these configurations, the section modules were calculated. On the basis of the modules, the diagrams presented in Figure 8a,b and Figure 9a,b were built. The single large dots represent the properties of the columns of the simulated inverted trapezoidal Kcer and trapezoidal Kcij configurations (Figure 8a,b). The Tin and Tout lines illustrate trends in changes in the values of the section module Smod of the column cross-sections caused by a change in their nc inclination, where nc is the ratio of d by the average height of the respective derivative frame, see Figure 3.

The single large dots shown in Figure 9a,b represent the properties of the diagonals of the inverted trapezial and trapezial configurations, respectively. The Tin and Tout lines illustrate the trends in changes in the optimal section modules of the cross-sections of the above diagonals caused by the change in the nc column slope.

Analysis of the properties of the lines shown in Figure 8 and Figure 9 allows one to observe the following relationships. There is a strong, significant influence of the nc inclination of the columns on their strength work, see the Tin and Tout lines in Figure 8a,b. The influence of column inclination on the strength performance of the diagonals of the derivative trapezial configurations is insignificant, see the lines Tin and Tout in Figure 9a,b.

The Tin and Tout lines presented in Figure 9a,b show the change in values of section modules calculated for the optimized cross-sections of the diagonals of the subsequently tested rectangular basic configuration Kb, derivative trapezial configurations Kcer for r = 1–4 (the Tin line), and derivative inverted trapezial configurations Kdij for j = 1–4 (the Tout line).

A significant qualitative tendency to changes in mechanical properties of the analyzed configurations can be observed in Figure 10, where the changes in the values of the critical load factor are caused by changes in the inclination of the columns to the vertical start with the value of about 1.0 and increase relatively fast. Due to the low value of the critical load factor φ_cr_ = 1.04 obtained during the optimizing process of the cross-sections of all elements belonging to the basic rectangular configuration Kb, the total load capacity of the vertical column’s cross-sections was not used. Therefore, the overall stability of the frame plays a decisive role in optimizing the Kb’s bars.

For all derived configurations Kci and Kce, the maximum load capacity of their columns is decisive. However, a greater ability to maintain overall stability is observed for the derivative trapezoidal configurations Kcer than for the inverted trapezoidal configurations Kcij. We can also say that the changes in the values of the φ_cr_ parameter non-linearly depend on the changes in the column inclination nc.

Thus, the innovative method for defining the specific loads of the considered frames supporting lower shelves of the folds of transformed roof sheeting, as loads distributed uniformly along the length of the upper chord of a roof frame girder, where all folds are supported by additional plates-tables fixed directly to the girder instead of using purlins transferring concentrated forces to the truss joints, was presented. The obtained results are the basis and justification for simulations and tests in the scope of further modification of the form, loads, work, and methods of using various configurations of flat frames.

## 7. Conclusions

There were presented unconventional premises resulting in the innovative topic of the research carried out in terms of checking the impact of changing the shape of subsequent flat frames intended for the construction of sheds (roofed with transformed thin-walled covers) on the geometric and mechanical properties of the members of these frames. The performed computer simulations confirmed the significance of the changes in shapes of the flat frame configurations from rectangular to trapezial resulting from the inclination of the columns to the vertical or the girders to the horizontal, making a significant increase in the effort of their elements working under the adopted types of loads.

For the defined loads and the adopted parameterization of the frame forms, an innovative set of conditions was developed to optimize their performance, and then a theoretical analysis of the observed dependencies was carried out. This analysis was performed in an unconventional, novel way using the section modules of the cross-sections of all members because the optimization of the cross-sections of the members of each analyzed base and derivative frame configuration had to be performed separately. The reason for such actions was the rapidly increasing effort of the elements with the increase in the inclination of the girder or columns when the arbitrary cross-sections of the initial derivative configurations were assumed to be identical to the optimized cross-sections of the base configuration calculated previously.

In the case of lattice girder inclination, a significant increase in the values of the section modules calculated for the optimized cross-sections of the shorter columns occurs. It can reach even 1000% for the most inclined girders. The significant increase in the section modules of the optimized bar cross-sections is also observed in the case of the bottom chord, where it reaches 400%. In the remaining optimized elements, the increase in the section modules of their cross-sections is insignificant.

In the case of the most tilted columns of the derivative frame configurations, the significant increase in the section modules of the optimized cross-sections is up to 200% for the trapezial configurations and 300% for the inverted trapezial configurations. In the case of other elements of these frames, the increase in effort is small.

The selected mechanical changes of the examined frame configurations caused by the changes in the inclination of their girders to the horizontal or their columns to the vertical are quantitative and qualitative. This results from the fact that the calculated critical loads and the ability to maintain the overall stability of the frames are the limitations determining the optimal size of the column’s cross-sections of the rectangular configuration. However, in the case of the considered trapezial and inverted trapezial frames, the decisive condition limiting the optimal size of the cross-sections of all their elements is the maximum allowable effort resulting from the yielding of the steel used. In the case of the derivative frame configurations, the values of critical load factor increase fast up to five and even seven for the frames with the most tilted elements.

## Figures and Tables

**Table 1:** The basic properties of the Kb’s elements calculated in the optimization process.

Element	Cross-Section [mm × mm]	Dp/dp Ratio	*σ_c_* [MPa]	*σ_t_* [MPa]	Displacement Δx_max_ [mm] Column P_s_	Deflection Δz_max_ [mm] Lattice Girder	Critical Load Factor φ_cr_
P_s_	177.8 × 4	44	207	−98			
P_d_	114.3 × 4	29	108	−236	11.4	33.0	1.04
P_g_	219.1 × 4.5	49	238	−130			
P_k_	114.3 × 3	38	157	−232			

**Table 2:** The compressive *σ_c_*/tensile *σ_t_* stresses calculated for the most stressed bars belonging to each type of the derivative configurations Kgi (i = 1–3).

Frame Configuration	Kg1	Kg2	Kg3
Frame Element	Cross−section [mm × mm]	Cross−section [mm × mm]	Cross−section [mm × mm]
P_s1_	139.7 × 4	168.3 × 4	188.8 × 4
P_s2_	355.6 × 5.6	457 × 6.3	457 × 6.3
P_d_	114.3 × 4.5	127 × 4.5	159 × 4
P_g_	244.5 × 4	219.1 × 5	244.5 × 4.5
P_k_	101.6 × 4	101.6 × 4	101.6 × 4
Stresses [MPa]	*σ_c_*/*σ_t_*	*σ_c_*/*σ_t_*	*σ_c_*/*σ_t_*
P_s1_	228/−122	237/−124	232/−136
P_s2_	217/−234	222/−230	218/−222
P_d_	125/−221	146/−218	165/−225
P_g_	228/−131	231/−136	225/−138
P_k_	156/−223	164/−229	198/−232
Displacement Δx_max_ [mm]	98.0	63.1	46.1
Deflection Δz_max_ [mm]	42.4	47.4	57.3
Critical load factor φ_cr_	7.07	5.81	5.76

**Table 3:** The compressive *σ_c_*/tensile *σ_t_* stresses calculated for the most stressed bars belonging to each element of the derivative configurations Kcij (j = 1–4).

Frame Configuration	Kci1	Kci2	Kci3	Kci4
Frame Element	Cross−section [mm × mm]	Cross−section [mm × mm]	Cross−section [mm × mm]	Cross−section [mm × mm]
P_s_	177.8 × 5.6	244.5 × 4	273 × 4.5	323.9 × 4.5
P_d_	114.3 × 4	114.3 × 4	114.3 × 4	114.3 × 4
P_g_	219.1 × 4.5	219.1 × 4.5	219.1 × 4.5	219.1 × 4.5
P_k_	101.6 × 3	108.3 × 3	88.9 × 3	76.1 × 3.6
Stresses [MPa]	*σ_c_*/*σ_t_*	*σ_c_*/*σ_t_*	*σ_c_*/*σ_t_*	*σ_c_*/*σ_t_*
P_s_	210/−121	218/−127	222/−148	214/−150
P_d_	179/−235	177/−233	195/−231	207/−228
P_g_	230/−131	217/−128	210/−129	228/−158
P_k_	179/−236	207/−233	209/−228	205/−224
Displacement x_max_ [mm]	187.0	143.7	147.0	132.5
Deflection z_max_ [mm]	32.1	33.1	32.0	31.0
Critical load factor φ_cr_	1.26	2.11	2.79	6.24

**Table 4:** The compressive *σ_c_*/tensile *σ_t_* stresses calculated for the most stressed bars belonging to each element of the derivative configurations Kcer (r = 1–4).

Frame Configuration	Kce1	Kce2	Kce3	Kce4
Frame Element	Cross−section [mm × mm]	Cross−section [mm × mm]	Cross−section [mm × mm]	Cross−section [mm × mm]
P_s_	177.8 × 4.5	193.7 × 4.5	193.7 × 5.6	273 × 5
P_d_	114.3 × 4	114.3 × 4	114.3 × 4	127 × 4.5
P_g_	244.5 × 4	244.5 × 4	244.5 × 4	273 × 4.5
P_k_	114.3 × 3	114.3 × 3	101.6 × 3	101.6 × 3
Stresses [MPa]	*σ_c_*/*σ_t_*	*σ_c_*/*σ_t_*	*σ_c_*/*σ_t_*	*σ_c_*/*σ_t_*
P_s_	211/−132	235/−160	233/−171	228/−153
P_d_	135/−236	140/−236	141/−236	129/−218
P_g_	222/−104	223/−104	225/−105	221/−91
P_k_	156/−223	176/−232	175/−226	206/−228
Displacement x_max_ [mm]	174.3	176.3	175.0	106.5
Deflection z_max_ [mm]	34.0	34.0	34.0	30.0
Critical load factor φ_cr_	1.23	1.29	2.02	4.37

## Data Availability

Data is available in tables presented in this article.

## Nomenclature

Kb	the optimized basic rectangular frame configuration
Kb0	the initial rectangular frame configuration optimized into Kb
Kg	the optimized derivative rectangular-trapezial frame configuration with inclined girder
Kce	the optimized derivative trapezial frame configuration (with inclined columns)
Kci	the optimized derivative inverted trapezial frame configuration (with inclined columns)
q	the load ununiformly distributed along the length of upper chord of girder
P_g_	the upper chord of a girder
P_d_	the lower chord of a girder
P_k_	the diagonal of a girder
P_s_	the column
Dp	the diameter of a pipe
dp	the thickness of a pipe
h	the difference between the length of two columns
d	the displacement of the column’s foundation
ng	the inclination of a girder
nc	the inclination of a column
*σ_c_*	the compressive stresses
*σ_t_*	the tensile stresses
φ_cr_	the critical load factor
Δx_max_	the maximum displacement in the x-axis direction
Δz_max_	the maximum deflection in the *z*-axis direction
[x,y,z]	the orthogonal coordinate system
(x, z)	one of three principal planes of [x,y,z].
